# Effect of Intravenous Morphine Injection on Neurological Examination of Dogs With Thoracolumbar Intervertebral Disk Extrusion

**DOI:** 10.3389/fvets.2020.571778

**Published:** 2020-10-16

**Authors:** Aurore Fouhety, Aurelie Bruwier, Jean Bassanino, Alexandra Gabriel, Jean-François Boursier, Anne-Sophie Bedu, Dimitri Leperlier

**Affiliations:** ^1^Centre Hospitalier Vétérinaire Pommery, Reims, France; ^2^Clinique Vétérinaire TouraineVet, Rochecorbon, France

**Keywords:** analgesia, modified frankel score, intervertebral disk disease, canine, morphine

## Abstract

**Objectives:** We aimed to determine the effect of intravenous morphine injection on the modified Frankel scores of dogs with thoracolumbar intervertebral disk extrusion (IVDE).

**Methods:** This was a prospective, blinded, randomized, and placebo-controlled study. We included dogs with a presumptive diagnosis of thoracolumbar IVDE that did not undergo analgesic, anti-inflammatory, or sedative treatment within the last 12 h. A neurological examination was performed and the deficits were graded using the modified Frankel score (MFS). Subsequently, each dog was randomly allocated to receive an intravenous injection of either morphine or placebo. After 30 min, the dogs were re-evaluated by the same veterinary officer who was blinded to the contents of the injections. Dogs were included in the study if IVDE was ultimately confirmed by surgery within one week of initial presentation.

**Results:** Among the 79 dogs initially enrolled, 62 dogs met the inclusion criteria. Among them, thirty-two dogs received intravenous morphine injections and there was no difference between the pre- and post-injection modified Frankel scores. Thirty dogs received an intravenous placebo injection. One dog had a worsening of the MFS by one grade in the post-injection examination.

**Clinical Significance:** In dogs with thoracolumbar intervertebral disk extrusion, an intravenous injection of morphine does not affect the modified Frankel score after 30 min compared with the pre-injection value. These findings support the use of an analgesic morphine dose if the neurological examination can be performed 30 min or later after the injection.

## Introduction

Thoracolumbar intervertebral disk extrusion (TL-IVDE) commonly affects dogs and its current treatment includes medical conservative management that involves rest, anti-inflammatory steroidal or non-steroidal and/or analgesic medication or surgery for spinal cord decompression (1–14).

The clinical signs of TL-IVDE may range from mild to severe neurological deficits associated with different discomfort and pain levels depending on the severity, type, and location of the disc extrusion (1, 7, 11, 13–16). Analgesic medication, including opioids, has to be considered for dogs with TL-IVDE. Given the speculated effect of opioids on neurological examination, their administration is frequently delayed until after the neurologic examination, since prognosis and surgical decisions are based on clinical neurological examination and scoring (6, 10–12, 17, 18). However, the consequences of using opioid drugs on neurological examination and scoring currently remain unclear.

We aimed to determine the effect of a single intravenous morphine injection on the modified Frankel scores (MFS) of dogs with subsequently confirmed TL-IVDE. We hypothesized that a single IV dose of 0.5 mg/Kg morphine would not affect the MFS.

## Materials and Methods

### Study Design and Setting

This prospective, blinded, randomized and placebo-controlled study was approved by the in-house ethical committee and informed owner consent was obtained in all cases.

### Neurological Examination

For each case involved in the present study a neurological examination was performed before and after the morphine injection.

The degree of spinal cord dysfunction was graded using a six-point MFS (19–23). Grade 0 corresponds to paraplegia without deep nociception, grade 1 to paraplegia without superficial nociception, grade 2 to paraplegia with nociception, grade 3 to nonambulatory paraparesis, grade 4 to ataxia or ambulatory paraparesis with loss or delayed proprioception, and grade 5 to spinal hyperesthesia only.

### Patient Selection and Randomization

Between January 2017 and December 2017, all dogs admitted with suspected TL-IVDE upon the first consultation were included. Dogs that received any analgesic, anti-inflammatory, or sedative drugs within the last 12 h were excluded. Subsequently, the included dogs underwent an initial complete neurological examination, which included gait assessment, cranial nerve evaluation, postural reaction testing, evaluation of nociception, and paraspinal palpation. Next, each dog randomly received a slow intravenous injection of either morphine or placebo (saline solution) based on a random function on an Excel file. Morphine (morphine chlorhydrate Cooper® Sanofi, Paris, France) was administered at a dosage of 0.5 mg/kg. Placebo was administered as an injection of an equivalent volume of saline solution. Injections were prepared and administered by a different clinician. Both injections were flushed using 1 mL of saline solution.

Thirty minutes after injection, another complete neurological examination was performed to determine the MFS. The same clinician, who was blinded to the administered substance, performed both neurological examinations. Dogs subsequently underwent pre- and post-contrast computed tomography (CT) under general anesthesia to determine the cause of neurologic deficits. A CT-myelography was also performed if it was necessary to confirm the diagnosis. Cases were definitively included in the study if a TL-IVDE diagnosis was made by diagnostic imaging and subsequently confirmed by surgery performed within one week of the imaging assessment. We excluded dogs with concomitant diseases that could interfere with hindlimb gait, diagnoses of other thoracolumbar diseases, and suspected TL- IVDE unconfirmed by surgery. For each included dog, we recorded the age, sex, breed, weight, complete neurological examination, pre- and post-injection MFS, the substance injected, and TL- IVDE site.

## Results

### Study Population

Seventy-nine dogs were initially included based on clinical suspicion of TL- IVDE, but only 62 met the inclusion criteria of the study. Three dogs were excluded that did not undergo the second neurological examination. Five dogs had a final diagnosis different from TL- IVDE (spinal cord neoplasia, suspected vertebral neoplasia, or suspected fibrocartilaginous embolism). One dog was excluded for having a concomitant cranial cruciate ligament rupture. Six dogs had a high suspicion of TL- IVDE according to diagnostic imaging; however, it was not confirmed by surgery. Finally, the owners of two dogs refused any investigation and opted for euthanasia.

The breeds of the included dogs were as follows: French bulldogs (*n* = 22), Dachshunds (*n* = 10), English cocker spaniels (*n* = 3), Yorkshire terriers (*n* = 4), Jack Russel terriers (*n* = 3), Bavarian dogs (*n* = 2), Cavalier King Charles spaniels (*n* = 2), Slovakian dogs (*n* = 2), mixed-breed dogs (*n* = 3), and one of each of the following breeds: German Shepherd dog, Coton de Tulear, Doguo Canario, Bernese mountain dog, Cane Corso, Pekingese dog, Pinscher, Shih Tzu, West Highland White Terrier, Dalmatian, and Pug. The median age and weight were 5.9 years (range: 2–13 years) and 13.6 kg (range: 2.7–68 kg), respectively. There were 27 intact male dogs, 17 intact female dogs, 6 castrated male dogs, and 12 neutered female dogs.

### Morphine Group

Thirty-two dogs received intravenous morphine injection. Among them 1, 3, 8, 6, 12, and 2 dogs were grade 0, 1, 2, 3, 4, and 5, respectively. There was no difference in the pre- and post-injection MFS for any of the dogs (Table 1).

**Table 1:** Modified Frankel score before and after injection summary, associated with the product injected.

**Number of case**	**MFS grade before injection**	**Product injected**	**MFS grade after injection**
1	4	Morphine	4
2	4	NaCl	4
3	4	NaCl	4
4	4	Morphine	4
5	4	Morphine	4
6	3	Morphine	3
7	4	NaCl	4
8	4	NaCl	4
9	4	Morphine	4
10	2	Morphine	2
11	3	NaCl	3
12	1	NaCl	1
13	2	NaCl	2
14	4	NaCl	4
15	5	Morphine	5
16	4	NaCl	4
17	0	Morphine	0
18	4	Morphine	4
19	4	NaCl	4
20	2	NaCl	2
21	2	NaCl	2
22	1	Morphine	1
23	4	NaCl	4
24	3	Morphine	3
25	1	Morphine	1
26	4	Morphine	4
27	4	NaCl	4
28	1	NaCl	1
29	4	Morphine	4
30	4	NaCl	4
31	2	Morphine	2
32	2	Morphine	2
33	2	Morphine	2
34	4	Morphine	4
35	1	NaCl	1
36	3	Morphine	3
37	2	NaCl	2
38	2	Morphine	2
39	4	NaCl	4
40	3	Morphine	3
41	4	NaCl	3
42	3	Morphine	3
43	1	NaCl	1
44	2	Morphine	2
45	2	NaCl	2
46	3	NaCl	3
47	4	Morphine	4
48	4	Morphine	4
49	4	Morphine	4
50	0	NaCl	0
51	3	Morphine	3
52	2	NaCl	2
53	4	Morphine	4
54	2	NaCl	2
55	2	Morphine	2
56	1	Morphine	1
57	4	NaCl	4
58	5	Morphine	5
59	2	NaCl	2
60	3	NaCl	3
61	2	Morphine	2
62	3	NaCl	3

### Placebo-Controlled Group

Thirty dogs received placebo. Among them, 1, 4, 8, 4, 13, and 0 dogs were grade 0, 1, 2, 3, 4, and 5, respectively. One dog in this group showed a different pre- and post-injection MFS grade, which worsened from grade 4 to 3 (Table 1).

## Discussion

In the present study, a single dose of IV morphine does not affect results of the neurological examination when results were graded using MFS. This finding has an important clinical relevance because all dogs with acute extruded disc have a lot of pain.

Opioids drugs, including morphine, are considered as the drug of choice for managing moderate to severe pain in dogs and cats (24). There are three different opioid receptors: mu, kappa, and delta. The analgesic morphine effect is mainly caused by its interaction with mu-opioid receptors (because of its high affinity for this particular receptor). However, interaction with the delta receptor can also induce mild to moderate analgesia (25, 26). This analgesia is mediated by direct stimulation of receptors mainly located in the brain and the dorsal horn of the spinal cord independent of metabolic activation (27, 28). However, some studies have suggested that opioid analgesics can produce analgesia without loss of awareness (27, 29).

Morphine has a very low oral bioavailability (> 20%) in dogs, which may be attributed to intestinal and hepatic presystemic metabolism (30). Intravenous morphine is commonly used for analgesia in dogs since it is safe and has few side effects. Vomiting due to delta receptor stimulation in the chemoreceptor trigger zone and histamine release can occur especially when intravenously administered too quickly (31–34). Intravenous morphine injection leads to a rapid increase in plasma drug levels and prompt analgesia and sedation (35). Plasma morphine levels decrease quickly, which results in a short effect duration (35, 36). Furthermore, morphine is among the cheapest opioids and is relatively accessible in our country, which justifies its use in the present study.

A study published in 2014, demonstrated the dose-dependent analgesic effect of morphine intravenously injected at 0.464, 0.681. and 1.0 mg/kg with all three dosages showing full antinociceptive efficiency (28). Plasma morphine levels > 20 ng/mL have an antinociceptive effect in dogs (35–37). This concentration is achieved between 5 and 60 min after intravenous morphine injection of 0.5 mg/kg (28, 35). Kögel reported that the peak effect was achieved at 10 min after the injection regardless of the intravenous morphine dosage (28). However, side effects, including sedation, salivation, and ataxia were observed at 0.681 mg/kg morphine while diarrhea, ataxia, salivation, and sedation were observed at 1.0 mg/kg (28). Ataxia and sedation are side effects of intravenous morphine injection that could interfere with neurological examination and grading. Therefore, according to previous findings, we administered an intravenous dosage of 0.5 mg/kg with a second neurological examination being performed 30 min after the injection (28).

By definition, a neurological examination is a subjective examination. To make it more objective and allow objective data comparison, we used an objective scale score. The MFS has been previously widely used in veterinary medicine to describe thoracolumbar spinal cord lesions (19–23, 38). There are other scales for grading spinal cord dysfunction, including the Texas Spinal Cord Injury Score or the 14-point pelvic limb neurologic score (22, 39). We used the MFS because it is easy to use in our daily practice and is probably the most commonly used scale for evaluating neurologic dysfunction; moreover, it has very low inter-observer variability (22). One dog had a worsen MFS score in the placebo group. This can be explained by a gradual evolution of the IVDE or its consequences on the spinal cord, assessed by the second neurological examination done 30 min later the first one.

We aimed to minimize any bias using a prospective, single-blinded observer, randomized, and placebo-controlled study design. However, this study has several limitations. We included dogs that received pain management treatment more than 12 h before their neurological assessment, since we assume that this analgesic, anti-inflammatory or sedative treatment would have similar effect on the two consecutive examinations.

Another limitation is that all neurological dysfunctions were not equitably represented, which could be attributed to our inclusion criteria. Dogs with a Grade 0 MFS were underrepresented in this study, because these cases were anesthetized quickly for CT after the initial examination and underwent emergency surgery before the second neurological examination. Moreover, many Grade 5 dogs were excluded of the protocol because these cases were medically managed, and so they did not have a surgically confirmed diagnosis of TL- IVDE. However, this resulting majority of grade 3 and 4 dogs is similar to that in large scale veterinary studies regarding TL- IVDE (22, 40).

## Conclusion

In conclusion, to our knowledge, this is the first prospective, blinded, randomized, and placebo-controlled study on the effect of intravenous morphine injection on neurological evaluation of dogs with TL- IVDE. We used intravenous morphine injection to obtain an analgesic effect on dogs with TL- IVDE, which did not affect the MFS after 30 min. These results support the immediate use of an analgesic dose of morphine even with the postponement of the neurological examination.

## Data Availability Statement

The original contributions presented in the study are included in the article/supplementary material, further inquiries can be directed to the corresponding author/s.

## Ethics Statement

The animal study was reviewed and approved by Ethics committee of CHV Pommery. Written informed consent was obtained from the owners for the participation of their animals in this study.

## Author Contributions

All authors listed have made a substantial, direct and intellectual contribution to the work. AF, J-FB, JB, AG, A-SB, and DL contributed to the conception and design of the study. AF, J-FB, and JB performed complete neurological examination and determined the MFS. J-FB and JB collected the data. AB and A-SB preformed pre- and post-injection contrast computed tomography and interpreted images. AF wrote the first draft of the manuscript. AF, AB, J-FB, JB, AG, A-SB, and DL contributed to the manuscript revision, read, and approved the submitted version.

## Conflict of Interest

The authors declare that the research was conducted in the absence of any commercial or financial relationships that could be construed as a potential conflict of interest.
